# Frequent emergency department utilization and syphilis case profiles in the Sentara healthcare system: a retrospective cross-sectional analysis in Hampton Roads

**DOI:** 10.1186/s12889-026-27785-4

**Published:** 2026-05-29

**Authors:** Martina Zamponi, Grace M. Tillotson, Chandler F. Knox, Mackenzie Tardif-Kunk, Thomas R. Campbell, Peter A. Mollica

**Affiliations:** 1 Eastern Virginia Medical School, Macon & Joan Brock Virginia Health Sciences at Old Dominion University, Norfolk, Virginia 23529 USA; 2 School of Medical Diagnostic and Translational Sciences, Ellmer College of Health Sciences, Macon & Joan Brock Virginia Health Sciences at Old Dominion University, Norfolk, Virginia 23529 USA; 3 School of Rehabilitation Sciences, Ellmer College of Health Sciences, Macon & Joan Brock Virginia Health Sciences at Old Dominion University, Norfolk, Virginia 23529 USA; 4 Molecular Diagnostics Laboratory, Sentara Norfolk General Hospital, Norfolk, Virginia 23529 USA

**Keywords:** Syphilis, Health disparities, Emergency department utilization, Healthcare access, Hampton Roads

## Abstract

**Background:**

Syphilis rates have increased substantially nationwide, disproportionately affecting marginalized populations and individuals with differing patterns of healthcare utilization. The Hampton Roads region of Virginia and North Carolina includes multiple jurisdictions with syphilis rates above the CDC screening threshold, with 7 of its 9 independent cities meeting this criterion. However, no prior study has examined the relationship between emergency department (ED) utilization patterns and documented syphilis diagnoses in this region.

**Methods:**

A retrospective cross-sectional analysis of the Sentara Healthcare network was conducted using aggregate data from the TriNetX platform. Patients diagnosed with syphilis between 2023 and 2024 were stratified by ED utilization frequency using two utilization thresholds: occasional ED users (< 4 or < 6 visits/year) and frequent ED users (≥ 4 or ≥ 6 visits/year). Demographic characteristics, syphilis stage distributions, and tertiary syphilis–related diagnoses were compared between groups using chi-square and normality testing.

**Results:**

Among 1,640 patients with documented syphilis diagnoses, 330 were classified as frequent ED users using the ≥ 4 ED visit threshold, and 170 using the ≥ 6 ED visit threshold. African American patients were significantly overrepresented in both frequent ED utilization groups compared with occasional ED users (69.69% vs. 56.48%, and 76.47% vs. 57.82%, respectively). Frequent ED users demonstrated higher proportions of documented late syphilis, latent late syphilis, and certain tertiary syphilis–related diagnoses. Overall differences in syphilis stage distribution and tertiary syphilis sequelae were more pronounced in sensitivity analyses using the ≥ 6 ED utilization threshold.

**Conclusions:**

Patients with higher levels of ED utilization demonstrated differing patterns of documented syphilis diagnoses, including higher proportions of certain late-stage and tertiary syphilis–related diagnoses, within this regional healthcare system. These findings may reflect differences in healthcare utilization patterns, screening practices, healthcare engagement, or diagnostic coding practices across patient populations. Further research using individual-level and longitudinal data is needed to better characterize factors associated with documented syphilis diagnosis patterns within the region.

**Supplementary Information:**

The online version contains supplementary material available at 10.1186/s12889-026-27785-4.

## Background

Syphilis is an infectious disease caused by the spirochete *Treponema pallidum*, which can be transmitted sexually or vertically (through pregnancy and birth). The natural progression of untreated syphilis includes symptomatic primary and secondary stages; the first characterized by the presence of nontender oral or genital lesions, and the second defined by the presence of systemic symptoms such as fever and maculopapular rash. The disease then enters an asymptomatic latent stage, which can last many years. In approximately 35% of cases, untreated late latent syphilis may progress to a newly symptomatic tertiary stage between 10 and 30 years from initial infection [1, 2]. Tertiary (late) syphilis can result in life-threatening complications including gummatous disease, neurosyphilis, and cardiovascular syphilis [3, 4].

Despite the long-established efficacy of penicillin as a treatment for syphilis [5], infection rates have been rising significantly and consistently since the early 2000s [6]. In 2023, there were 209,253 cases of syphilis reported nationwide, with an overall increase of 1.0% in total diagnoses compared to the previous year [7]. In Virginia, the year-over-year increase in diagnoses between 2022 and 2023 amounted to 22% [8]. One major driver of syphilis transmission is the prevalence of infection within the local community. To mitigate this spread, the CDC recommends offering syphilis testing to all sexually active individuals between the ages of 15–44 in counties where the rate of infection is higher than 4.6 per 100,000 people. While the optimal screening frequency for the general population or for individuals residing in high-prevalence regions is not explicitly defined, current guidelines recommend at least annual screening for individuals at increased risk, with more frequent testing when risk persists [9–11]. Of particular interest is the Hampton Roads region, where the syphilis rate warrants screening in 7 of the 9 independent cities included in this geographical area [7]. Detailed syphilis rate information is presented in Table 1. Hampton Roads is a coastal metropolitan region of southeastern Virginia and northeastern North Carolina with a population of approximately 1.5 million people, according to the most recent U.S. Census Bureau American Community Survey 5-year estimates [12]. Based on these estimates, the population is approximately 50% White, 33% Black, 4% Asian, and 13% other races. A detailed breakdown of the racial distribution across the nine independent cities included in the study region is provided in Supplementary Table S1. The region is demographically diverse, influenced in part by its large military presence, major maritime industries, and historically established African American communities.

**Table 1:** Syphilis rate per 100,000 people in the independent cities of Hampton Roads region in Virginia

City	Rate per 100,000
Virginia Beach	1.1
Norfolk	17.1
Chesapeake	7.8
Newport News	5
Hampton	17
Portsmouth	38.7
Suffolk	34.9
Williamsburg	40.7
Poquoson	0

Social determinants of health (SDOH), including low income, lack of health insurance, limited access to healthcare, and racial/ethnic minority status, have been consistently linked to increased rates of sexually transmitted infections [13–16]. Particularly, syphilis disproportionately affects patients of color and those with limited access to primary care [7]. Syphilis testing for early detection and treatment is a critical component of infection control and prevention of late-stage disease complications. The importance of implementing syphilis screening measures as a public health intervention to address the current state of syphilis prevalence has been recognized by various agencies [17].

Vulnerable populations disproportionately affected by syphilis may also experience differing patterns of healthcare utilization and barriers to appropriate testing and treatment. Prior studies have demonstrated associations between reduced primary care access and increased emergency department (ED) utilization [18–21]. Consequently, frequency of ED utilization may serve as a marker of differing healthcare utilization patterns and broader social determinants of health.

Understanding the current state of syphilis case profiles within the Hampton Roads community is crucial to better understand patient needs and inform future public health efforts. To the best of our knowledge, no study has specifically evaluated syphilis case characteristics in socioeconomically vulnerable populations within this region. Here we investigate the syphilis case profiles and documented disease characteristics among frequent ED users in the Hampton Roads region, used as a proxy indicator for potential barriers to primary care access.

## Methods

### Data acquisition and group construction

This retrospective cross-sectional study was conducted using aggregate data from the TriNetX Sentara network. Electronic Health Record (EHR) data from over 4 million patients were collected within the Sentara Healthcare Organization (HCO). This database was chosen as the focus of this study due to its dominant presence within the Hampton Roads region, encompassing 12 acute care hospitals and over 300 sites of care, therefore providing a comprehensive representation of the local patient population.

Patients of all ages with a documented diagnosis of syphilis between the years 2023 and 2024 were identified using the following ICD-10 codes: A51, A51.0, A51.1, A51.2, A51.3, A51.39, A51.4, A51.42, A51.43, A51.45, A51.49, A51.5, A51.9, A52, A52.0, A52.00, A52.03, A52.05, A52.09, A52.1, A52.11, A52.12, A52.13, A52.16, A52.17, A52.3, A52.31, A52.7, A52.72, A52.73, A52.74, A52.76, A52.78, A52.79, A52.8, A52.9, A53, A53.0, A53.9. A detailed description of the diagnosis associated with each ICD-10 code is provided in Supplementary Table S2. Syphilis-related ICD-10 codes are not mutually exclusive; patients may receive more than one syphilis diagnosis code, which results in category totals exceeding the number of unique individuals. When a time constraint is applied within the TriNetX platform, diagnosis encounters must occur within the selected timeframe to be included in the cohort definition. Therefore, only patients with a documented syphilis diagnosis recorded in either 2023 or 2024 were included in this study. Patients with congenital syphilis were excluded.

Two sets of comparison groups were created based on emergency department (ED) utilization, determined using the CPT code 1,013,711 or the visit type designation “Visit: Emergency”. In the primary analysis, the Occasional ED Users group included patients who visited the ED fewer than 4 times per year, while the Frequent ED Users group included patients who utilized ED services 4 or more times in a year. A secondary analysis was also performed using a more stringent cutoff, defining Frequent ED Users as patients with 6 or more ED visits per year and Occasional ED Users as patients with fewer than 6 ED visits per year. While there is no universally established guideline defining a frequent ED user, the cutoff of four or more visits within a 12-month period is widely used in emergency medicine and health services research [22–25]. Frequent ED use was used as a marker of differing healthcare utilization patterns, based on prior literature demonstrating associations between ED reliance, healthcare access, and broader social determinants of health [18–21].

Basic demographic data were collected, including sex, age, race, ethnicity and HIV status. Syphilis stage and tertiary complications were identified using the ICD-10 codes listed in Supplementary Table S2, with no restriction on where the diagnosis was made (i.e., diagnoses were not limited to ED encounters). HIV status was determined using the ICD-10 code B20 (Human immunodeficiency virus disease), which was used to identify the proportion of patients in each cohort with a documented HIV diagnosis. Secondary stratified analyses of tertiary syphilis complications were subsequently performed separately among HIV-positive and HIV-negative patients to evaluate whether observed differences persisted after accounting for HIV status.

Within the TriNetX platform, groups were created by defining a set of inclusion and exclusion criteria—in this case, syphilis diagnosis, emergency department (ED) utilization frequency, and HIV status for the stratified analyses. Once these criteria were applied, TriNetX queried the Sentara network database and identified all patients who met the specified conditions, thereby generating the study groups. After the groups were established, the platform provided aggregated data describing the patients within each group. These aggregate outputs include, but are not limited to, demographics, diagnoses, procedures, medications, and laboratory values. For example, once a group is defined, TriNetX can report the number and percentage of patients within that group who have a given characteristic, such as a specific diagnosis, demographic attribute, or laboratory result.

All data obtained from TriNetX were aggregated and de-identified. No individual-level patient charts or line-level variables were available at any point. Because the dataset includes only encounters within the Sentara Healthcare system, both syphilis diagnoses and ED utilization reflect only patients who sought care within this system. As a result, we were unable to determine what proportion of all syphilis cases in the Hampton Roads region are represented in this dataset.

### Data analysis

Normality of age distributions in the two ED utilization groups was assessed using standard tests (D’Agostino–Pearson, Anderson–Darling, Shapiro–Wilk, and Kolmogorov–Smirnov). For demographic characteristics, syphilis stages, and tertiary complications, the number and proportion of patients in each group were calculated. TriNetX rounds reported counts as part of its de-identification procedures; therefore, category-level counts may not be mutually exclusive or sum exactly to group totals. In addition, the primary analysis (≥ 4 ED visits), secondary analysis (≥ 6 ED visits), and HIV-stratified analyses were conducted as separate TriNetX queries. Due to TriNetX de-identification and rounding practices, aggregate values generated from separate analyses may not directly correspond with one another, even when derived from the same underlying patient population.

Group differences in proportions between Frequent and Occasional ED users were initially assessed using chi-square tests. A two-sided p-value < 0.05 was considered statistically significant. When the overall chi-square comparison was statistically significant, individual diagnostic categories were subsequently compared using chi-square testing with Bonferroni correction for multiple comparisons. Individual category-level p-values were also displayed in instances where the overall comparison was not statistically significant for descriptive purposes. All statistical analyses were performed using GraphPad Prism version 10.4.2 for Windows (GraphPad Software, Boston, Massachusetts, USA).

### IRB approval

This retrospective cross-sectional study is exempt from Institutional Review Board approval as the data reviewed is a secondary analysis of existing data, does not involve intervention or interaction with human subjects, and is de-identified per the de-identification standard defined in Section § 164.514(a) of the HIPAA Privacy Rule.

## Results

This retrospective cross-sectional study describes syphilis case profiles among patients diagnosed within the Sentara Healthcare system in the Hampton Roads region, comparing frequent and occasional ED users to evaluate differences across varying levels of ED utilization.

### Demographics

A total of 1,640 patients diagnosed with syphilis within the Sentara network between 2023 and 2024 were identified.

Within the ≥ 4 ED utilization analysis, ages ranged between 18 and 90 years among occasional ED users, with a mean age of 47 years (standard deviation [SD] 17), and 50.8% of patients falling within the CDC’s recommended screening age range of 15–44 years. Among frequent ED users, ages ranged between 18 and 88 years, with a mean age of 46 years (SD 17), and 55.16% of the cohort falling between 15 and 44 years. The age distribution was assessed in both groups; the occasional ED group demonstrated a normal distribution across all tests performed, whereas the frequent ED group did not.

The proportion of male versus female patients was slightly higher in the occasional ED group compared with the frequent ED groups – 65.64% vs. 63.63%, and 65.30% vs. 58.82%.

In both study groups, most patients were African American, representing 56.48% of occasional ED users and 69.69% of frequent ED users, within the ≥ 4 ED utilization group. Conversely, the proportion of White patients (34.35% vs. 27.27%) was lower in the frequent ED group.

Within the secondary ≥ 6 ED utilization analysis, demographic patterns remained generally similar. Frequent ED users demonstrated a slightly older mean age compared with the ≥ 4 cohort (50 years, SD 17), while occasional ED users maintained a mean age of 47 years (SD 17). African American patients continued to represent the majority of both groups, accounting for 76.47% of frequent ED users and 57.82% of occasional ED users. HIV status was similar between groups across both ED utilization thresholds. Detailed demographic characteristics for both analyses are presented in Table 2.

**Table 2:** Demographic characteristics of patients with documented syphilis diagnoses by ED utilization group

Characteristic	≥ 4 ED Frequent (*n* = 330)	≥ 4 ED Occasional (*n* = 1310)	≥ 6 ED Frequent (*n* = 170)	≥ 6 ED Occasional (*n* = 1470)
Sex				
Male	63.63%	65.64%	58.82%	65.30%
Female	39.39%	35.11%	41.18%	35.37%
Age, mean (SD)	47 (17)	47 (17)	50 (17)	47 (17)
Race				
African American	69.69%	56.48%	76.47%	57.82%
White	27.27%	34.35%	23.52%	34.01%
Asian	3.03%	1.52%	0.00%	1.36%
Other	9.09%	9.91%	11.76%	8.84%
HIV Status				
Positive	100 (30.0%)	400 (31.0%)	50 (29.0%)	450 (31.0%)
Negative or unknown	230 (70.0%)	910 (69.0%)	120 (71.0%)	1020 (69.0%)

Values represent counts and percentages of patients within each ED utilization group, unless otherwise indicated. Due to rounding and the aggregate reporting structure of TriNetX, demographic category totals may not sum exactly to 100%

### Syphilis stages

The distribution of syphilis stages across both study groups was assessed using ICD-10 diagnosis codes. Overall stage distribution did not significantly differ between frequent and occasional ED users in the ≥ 4 ED utilization analysis (χ²(7) = 8.000, *p* = 0.3326). However, in the secondary ≥ 6 ED utilization analysis, overall stage distribution differed significantly between groups (χ²(7) = 33.79, *p* < 0.0001).

Descriptively, frequent ED users demonstrated higher proportions of primary genital syphilis (6.5% vs. 4.0%), late syphilis (32.3% vs. 24.8%), and latent late syphilis (12.9% vs. 8.8%) compared with occasional ED users in the ≥ 4 ED utilization analysis. However, these subgroup comparisons were exploratory and should be interpreted cautiously, as the overall stage distribution was not statistically significant.

Within the ≥ 6 ED utilization analysis, frequent ED users similarly demonstrated higher proportions of late syphilis (35.3% vs. 24.5%) and latent late syphilis (23.5% vs. 8.8%) compared with occasional ED users, and these differences remained statistically significant following Bonferroni correction for multiple comparisons. Proportions of early latent syphilis and secondary syphilis were similar between groups. Full descriptive stage comparisons are presented in Table 3. Bonferroni-corrected statistical significance was defined as *p* < 0.00625 (0.05/8 comparisons). Asterisks indicate comparisons remaining statistically significant after correction. *P*-values for individual subgroup comparisons are also displayed for descriptive purposes.

**Table 3:** Differences in syphilis stage of diagnosis between occasional and frequent ED users

Diagnosis	≥ 4 ED Frequent (*n* = 330)	≥ 4 ED Occasional (*n* = 1310)	*P*-value	≥ 6 ED Frequent (*n* = 170)	≥ 6 ED Occasional (*n* = 1470)	*P*-value
Early syphilis	50 (15.0%)	210 (16.0%)	0.6960	20 (11.8%)	240 (16.3%)	0.123
Primary genital syphilis	20 (6.0%)	50 (4.0%)	0.0402	10 (5.8%)	60 (4.1%)	0.272
Secondary syphilis of skin and mucous membranes	10 (3.0%)	50 (4.0%)	0.6203	10 (5.8%)	50 (3.4%)	0.103
Early syphilis, latent	10 (3.0%)	40 (3.0%)	0.9826	10 (5.8%)	50 (3.4%)	0.103
Late syphilis	100 (30.0%)	320 (24.0%)	0.0047	60 (35.3%)	360 (24.5%)	0.0022 *
Late syphilis, latent	40 (12.0%)	120 (9.0%)	0.0470	40 (23.5%)	130 (8.8%)	< 0.0001 *
Latent syphilis, unspecified as early or late	100 (30.0%)	360 (27.0%)	0.1793	60 (35.3%)	410 (27.9%)	0.043
Other and unspecified syphilis	270 (82.0%)	1,080 (82.0%)	0.7904	140 (82.4%)	1210 (82.3%)	0.990

Overall stage distribution differed significantly between groups in the ≥ 6 ED utilization analysis but not in the ≥ 4 ED utilization analysis. Bonferroni-corrected statistical significance for individual comparisons was defined as *p* < 0.00625 (0.05/8 comparisons)

Asterisks indicate comparisons remaining statistically significant after correction. Individual subgroup *p*-values are also displayed for descriptive purposes

### Tertiary syphilis sequelae

The distribution of tertiary syphilis-related diagnoses across sequelae categories was assessed using ICD-10 diagnosis codes. Overall distribution of tertiary syphilis sequelae did not significantly differ between frequent and occasional ED users in the ≥ 4 ED utilization analysis (χ²(4) = 6.543, *p* = 0.1621). However, in the secondary ≥ 6 ED utilization analysis, overall sequelae distribution differed significantly between groups (χ²(4) = 18.97, *p* = 0.0008).

Within the ≥ 4 ED utilization analysis, frequent ED users demonstrated a higher proportion of symptomatic neurosyphilis (12.0% vs. 7.0%) and asymptomatic neurosyphilis (3.0% vs. 1.0%) diagnoses compared with occasional ED users. Other tertiary syphilis-related diagnoses, including cardiovascular/cerebrovascular syphilis, symptomatic neurosyphilis, neurosyphilis unspecified, and other symptomatic late syphilis, were proportionally similar between groups. Because the overall sequelae distribution was not statistically significant in the ≥ 4 analysis, these subgroup comparisons should be interpreted cautiously and are presented for descriptive purposes.

Within the ≥ 6 ED utilization analysis, frequent ED users demonstrated higher proportions of cardiovascular/cerebrovascular syphilis (5.8% vs. 1.4%) and asymptomatic neurosyphilis (5.8% vs. 0.7%) compared with occasional ED users, and these differences remained statistically significant following Bonferroni correction for multiple comparisons. Full descriptive comparisons are presented in Tables 4, 5 and 6.

**Table 4:** Differences in late syphilis complications between occasional and frequent ED users

Diagnosis	≥ 4 ED Frequent (*n* = 330)	≥ 4 ED Occasional (*n* = 1310)	*P*-value	≥ 6 ED Frequent (*n* = 170)	≥ 6 ED Occasional (*n* = 1470)	*P*-value
Cardiovascular and cerebrovascular syphilis	10 (3.0%)	20 (2.0%)	0.0685	10 (5.8%)	20 (1.4%)	< 0.0001 *
Symptomatic neurosyphilis	40 (12.0%)	90 (7.0%)	0.0016	20 (11.8%)	110 (7.5%)	0.050
Asymptomatic neurosyphilis	10 (3.0%)	10 (1.0%)	0.0004	10 (5.8%)	10 (0.7%)	< 0.0001 *
Neurosyphilis, unspecified	40 (12.0%)	100 (8.0%)	0.0091	20 (11.8%)	110 (7.5%)	0.050
Other symptomatic late syphilis	10 (3.0%)	40 (3.0%)	0.9826	10 (5.8%)	50 (3.4%)	0.103

Overall sequelae distribution differed significantly between groups in the ≥ 6 ED utilization analysis but not in the ≥ 4 ED utilization analysis. Bonferroni-corrected statistical significance for individual comparisons was defined as *p* < 0.01 (0.05/5 comparisons)

Asterisks indicate comparisons remaining statistically significant after correction. Individual subgroup *p*-values are also displayed for descriptive purposes

**Table 5:** Differences in late syphilis complications between HIV-positive occasional and frequent ED users

Diagnosis	≥ 4 ED Frequent (*n* = 100)	≥ 4 ED Occasional (*n* = 390)	*P*-value	≥ 6 ED Frequent (*n* = 50)	≥ 6 ED Occasional (*n* = 430)	*P*-value
Cardiovascular and cerebrovascular syphilis	0 (0.00%)	10 (3.0%)	0.1057	0 (0.00%)	10 (2.0%)	0.1237
Symptomatic neurosyphilis	10 (10.0%)	10 (3.0%)	0.0008 *	10 (20.0%)	10 (2.0%)	< 0.0001 *
Asymptomatic neurosyphilis	10 (10.0%)	10 (3.0%)	0.0008 *	10 (20.0%)	10 (2.0%)	< 0.0001 *
Neurosyphilis, unspecified	20 (20.0%)	50 (13.0%)	0.0672	10 (20%)	50 (12.0%)	0.0902
Other symptomatic late syphilis	10 (10.0%)	20 (5.0%)	0.0698	10 (20.0%)	20 (5.0%)	< 0.0001 *

Overall sequelae distribution differed significantly between groups in both the ≥ 4 and ≥ 6 ED utilization analyses among HIV-positive patients. Bonferroni-corrected statistical significance for individual comparisons was defined as *p* < 0.01 (0.05/5 comparisons)

Asterisks indicate comparisons remaining statistically significant after correction. Individual subgroup *p*-values are also displayed for descriptive purposes

**Table 6:** Differences in late syphilis complications between HIV-negative occasional and frequent ED users

Diagnosis	≥ 4 ED Frequent (*n* = 240)	≥ 4 ED Occasional (*n* = 930)	*P*-value	≥ 6 ED Frequent (*n* = 120)	≥ 6 ED Occasional (*n* = 1050)	*P*-value
Cardiovascular and cerebrovascular syphilis	10 (4.0%)	20 (2.0%)	0.0781	10 (8.0%)	20 (2.0%)	< 0.0001
Symptomatic neurosyphilis	30 (13.0%)	90 (10.0%)	0.1988	20 (17.0%)	100 (9.0%)	0.0146
Asymptomatic neurosyphilis	0 (0.0%)	0 (0.0%)	na	0 (0.0%)	0 (0.0%)	na
Neurosyphilis, unspecified	20 (8.0%)	50 (5.0%)	0.0851	10 (8.0%)	60 (6.0%)	0.2518
Other symptomatic late syphilis	10 (4.0%)	30 (3.0%)	0.4745	10 (8.0%)	30 (3.0%)	0.0018

Overall sequelae distribution did not significantly differ between groups in either the ≥ 4 or ≥ 6 ED utilization analyses among HIV-negative patients. Bonferroni-corrected statistical significance for individual comparisons was defined as *p* < 0.01 (0.05/5 comparisons)

Asterisks indicate comparisons remaining statistically significant after correction. Individual subgroup *p*-values are also displayed for descriptive purposes

Among HIV-positive patients, overall tertiary syphilis sequelae distribution differed significantly between frequent and occasional ED users in both the ≥ 4 ED utilization analysis (χ²(4) = 10.71, *p* = 0.030) and the ≥ 6 ED utilization analysis (χ²(4) = 17.50, *p* = 0.0015). In contrast, among HIV-negative patients, overall sequelae distribution did not significantly differ between groups in either the ≥ 4 ED utilization analysis (χ²(3) = 1.024, *p* = 0.7954) or the ≥ 6 ED utilization analysis (χ²(3) = 6.308, *p* = 0.0975).

## Discussion

Previous studies have shown that social determinants of health and lack of appropriate healthcare access contribute to disproportionate syphilis burden in certain populations. To our knowledge, this is the first study to describe syphilis case profiles among frequent emergency department users within the Sentara Healthcare system in the Hampton Roads region. ED utilization was used as a marker of differing healthcare utilization patterns, based on prior literature demonstrating associations between ED reliance, healthcare access, and broader social determinants of health. The timeframe of 2023–2024 was selected to provide an updated characterization of syphilis diagnoses while minimizing potential confounding from the COVID-19 pandemic. Overall, we found that frequent ED users, defined as patients with four or more ED visits in a year, had higher proportions of documented late syphilis diagnoses and certain tertiary syphilis–related ICD codes compared with occasional ED users.

Current CDC recommendations include syphilis screening for all sexually active patients between the ages of 15–44 in regions where syphilis rates are above 4.6 per 100,000 people. This includes the Virginian cities of Norfolk, Chesapeake, Newport News, Hampton, Portsmouth, Suffolk and Williamsburg. In our analysis, approximately half of occasional ED users diagnosed with syphilis fell within this recommended screening age range. A somewhat larger proportion of frequent ED users were also within this age range. While we observed differences in the age ranges between the two ED utilization groups, these findings should be interpreted cautiously, as TriNetX does not allow for the analysis of individual-level data. The pattern we observed may reflect existing screening practices within Sentara health [26], but alternative explanations—including underlying demographic differences in healthcare-seeking behavior—cannot be excluded.

With respect to demographic characteristics, men represented a slightly higher proportion of syphilis diagnoses compared with women in both ED utilization groups. African American patients represented a significantly larger proportion of frequent ED users compared with occasional ED users. While race is not itself a risk factor for syphilis and is not always associated with increased STI incidence [14], our findings align with national reports documenting a disproportionate burden of STIs among racial and ethnic minority populations. In the Hampton Roads region, African Americans constitute approximately 33% of the population according to U.S. Census estimates; however, they represented 56% of occasional ED users with a syphilis diagnosis and 74–76% of frequent ED users in this study, indicating a disproportionately higher burden of infection in this population.

Frequent ED users demonstrated proportionally higher rates of certain later-stage and tertiary syphilis-related diagnoses, including late syphilis, latent late syphilis, and asymptomatic neurosyphilis. In the primary ≥ 4 ED utilization analysis, overall distributions of syphilis stage and tertiary syphilis sequelae did not significantly differ between frequent and occasional ED users, suggesting that while some descriptive subgroup trends were observed, broader stage progression patterns were generally similar across groups. However, secondary sensitivity analyses using a more stringent ≥ 6 ED visit threshold demonstrated significant overall differences in both syphilis stage distribution and tertiary syphilis-related sequelae between groups. Importantly, the directionality of observed subgroup trends remained generally consistent across both ED utilization thresholds, with stronger associations observed at higher levels of ED utilization. These findings may reflect differences in healthcare utilization patterns, screening practices, delayed diagnosis, or diagnostic coding practices among patients with differing levels of ED utilization, but should be interpreted cautiously given the aggregate and observational nature of the dataset.

HIV status represents an important potential confounder in the interpretation of late-stage and tertiary syphilis diagnoses, particularly neurosyphilis-related diagnoses. HIV-positive patients may undergo more frequent syphilis screening, neurologic evaluation, and cerebrospinal fluid testing, which could increase the likelihood of detecting asymptomatic or late-stage disease [27, 28]. To further evaluate this potential confounding effect, secondary stratified analyses were performed separately among HIV-positive and HIV-negative patients. Among HIV-positive patients, overall tertiary syphilis sequelae distributions differed significantly between frequent and occasional ED users at both ED utilization thresholds, whereas no significant overall differences were observed among HIV-negative patients. These findings suggest that differences in tertiary syphilis-related diagnoses may be influenced in part by differences in HIV-associated screening and diagnostic practices. Additionally, men who have sex with men (MSM) represent one of the populations most disproportionately affected by syphilis in the United States and also demonstrate higher rates of HIV coinfection and STI screening, which may further influence patterns of documented syphilis diagnoses within this population [29, 30]. However, given the aggregate nature of TriNetX data and the inability to assess individual diagnostic workups or temporal relationships between diagnoses, these findings should be interpreted cautiously and considered hypothesis-generating.

Overall, this study identified differences in patterns of documented syphilis diagnoses among patients with differing levels of ED utilization within the Sentara Healthcare system. Importantly, syphilis diagnoses and tertiary syphilis-related ICD codes were identified across the Sentara Healthcare system and were not restricted to ED encounters themselves; ED utilization was used only to define comparison groups based on patterns of healthcare utilization. These findings highlight the potential value of considering factors associated with social determinants of health and healthcare utilization when refining public health approaches for syphilis detection. Observed differences may reflect variation in healthcare utilization patterns, screening practices, healthcare engagement, or diagnostic coding practices across patient populations. However, given the aggregate and observational nature of the dataset, these findings should be interpreted cautiously and should not be interpreted as evidence of causal relationships at the individual patient level. Future research using individual-level data, longitudinal follow-up, and more detailed social and behavioral variables is needed to further characterize factors associated with syphilis diagnosis patterns within the region.

### Limitations

This retrospective cross-sectional study presents several limitations associated with its design, the data platform utilized, and characteristics of the dataset.

First, due to the study’s observational and cross-sectional nature, it is not possible to establish a definitive causal and temporal relationship between healthcare access, ED utilization, and the distribution of documented syphilis diagnoses. Likewise, the data do not allow assessment of syphilis incidence, prevalence, or clinical progression [31].

Second, the use of aggregated, de-identified data from the TriNetX platform, while providing access to a large quantity of data, limits the granularity of the information available for analysis It is not possible to access individual-level variables—such as insurance status, ZIP code or census tract, prior healthcare utilization, comorbidities, socioeconomic factors, sexual behaviors, MSM status, or detailed clinical characteristics. Importantly, frequent ED utilization can reflect multiple drivers beyond limited primary care access—including substance use disorders, psychiatric comorbidity, housing instability/homelessness, and proximity or reliance on ED-based services—and these factors cannot be disentangled in this aggregate dataset. Consequently, confounding related to unmeasured social determinants of health cannot be excluded. Additionally, the dataset does not provide information regarding syphilis screening practices, cerebrospinal fluid testing, neurologic evaluations, or sexual behavior risk factors, all of which may influence the likelihood of documenting late-stage or tertiary syphilis diagnoses. Consequently, these findings should be interpreted as population-level associations rather than evidence of causal relationships at the individual patient level. Further, because TriNetX reports rounded aggregate counts to protect individual-level data, category totals may not sum precisely to group totals. For the same reason, it is not possible to directly link each syphilis diagnosis to laboratory confirmation within the same encounter.

Third, our dataset was limited to encounters within the Sentara Healthcare system. Patients who received syphilis testing, diagnosis or treatment outside this system may not be represented. Therefore, we were unable to establish the proportion of all syphilis cases in the Hampton Roads region represented by this dataset, and the findings cannot be interpreted as population-level estimates.

Finally, this retrospective study relied on the utilization of ICD-10 diagnosis codes to identify syphilis stages and tertiary syphilis–related conditions. The choice of ICD-10 diagnosis codes is largely dependent on individual physicians’ preferences and practices. Moreover, ICD-10 codes are not mutually exclusive, and patients may receive multiple ICD-10 diagnosis codes related to the same condition. These factors limit the ability to interpret ICD codes as precise clinical staging or indicators of disease severity or progression.

## Conclusion

In this retrospective cross-sectional analysis, frequent ED users within the Sentara Healthcare system had higher proportions of documented late and tertiary syphilis–related ICD-10 codes compared with occasional ED users. These findings should be interpreted as preliminary and hypothesis-generating, given the aggregate nature of the data and the potential for unmeasured confounding. African American patients represented a disproportionately large portion of both frequent and occasional ED users, consistent with broader inequities observed in national STI surveillance. Our findings underscore the differences in documented syphilis diagnoses among patients with varying levels of ED utilization, highlighting the potential relevance of healthcare access indicators when considering syphilis screening approaches. Additional research incorporating individual-level and longitudinal data is needed to clarify the factors contributing to these patterns and to evaluate whether access-related indicators may have a role in future public health strategies. 

## Supplementary Information

Additional file 1: Table S1. Racial composition of the population of Hampton Roads cities included in the study region (U.S. Census Bureau American Community Survey 5-year estimates).

Additional file 2: Table S2. ICD-10-CM codes utilized to identify syphilis diagnosis and sequelae.

## Data Availability

The data that support the findings of this study are available from TriNetX and Sentara Health, but restrictions apply to the availability of these data, which were used under license for the current study, and so are not publicly available. Data are, however, available from the authors upon reasonable request and with permission of Sentara Health.
