# Supplementary material for: Frequent emergency department utilization and syphilis case profiles in the Sentara healthcare system: a retrospective cross-sectional analysis in Hampton Roads
Source: BMC Public Health. 2026 May 29;26:2230. doi: 10.1186/s12889-026-27785-4 (PMC13411110; doi:10.1186/s12889-026-27785-4)
Supplement: Supplementary file 1 — Additional file 1: Table S1. Racial composition of the population of Hampton Roads cities included in the study region (U.S. Census Bureau American Community Survey 5-year estimates). [file 12889_2026_27785_MOESM1_ESM.docx]

**Additional File 1. Table S1.** Racial composition of the population of Hampton Roads cities included in the study region (U.S. Census Bureau American Community Survey 5-year estimates).

| **City** | **Population** | **White** | **Black** | **Asian** | **Other** |
| --- | --- | --- | --- | --- | --- |
| Norfolk | 231,105 | 43.2% | 39.6% | 3.7% | 13.5% |
| Virginia Beach | 454,808 | 60.8% | 18.8% | 7.2% | 13.2% |
| Chesapeake | 254,997 | 55.1% | 29.1% | 3.9% | 11.9% |
| Newport News | 183,056 | 41.0% | 41.2% | 3.3% | 14.5% |
| Portsmouth | 96,482 | 36.2% | 50.6% | 1.7% | 11.5% |
| Hampton | 137,596 | 36.7% | 49.3% | 2.3% | 11.7% |
| Suffolk | 103,105 | 46.5% | 42.0% | 1.9% | 9.6% |
| Williamsburg | 16,030 | 68.1% | 14.4% | 6.8% | 10.7% |
| Poquoson | 12,854 | 90.1% | 1.9% | 1.7% | 6.3% |
|  |  |  |  |  |  |
| Hampton Roads | 1,490,033 | 50.2% | 32.8% | 4.4% | 12.6% |

Percentages were calculated using population-weighted estimates across cities.
