# Supplementary material for: Frequent emergency department utilization and syphilis case profiles in the Sentara healthcare system: a retrospective cross-sectional analysis in Hampton Roads
Source: BMC Public Health. 2026 May 29;26:2230. doi: 10.1186/s12889-026-27785-4 (PMC13411110; doi:10.1186/s12889-026-27785-4)
Supplement: Supplementary file 2 — Additional file 2: Table S2. ICD-10-CM codes utilized to identify syphilis diagnosis and sequelae. [file 12889_2026_27785_MOESM2_ESM.docx]

**Additional File 2. Table S2.** ICD-10-CM codes utilized to identify syphilis diagnosis and sequelae.

| **ICD-10-CM Code** | **Diagnosis** |
| --- | --- |
| A51 | Early syphilis |
| A51.0 | Primary genital syphilis |
| A51.1 | Primary anal syphilis |
| A51.2 | Primary syphilis of other sites |
| A51.3 | Secondary syphilis of skin and mucous membranes |
| A51.39 | Other secondary syphilis of skin |
| A51.4 | Other secondary syphilis |
| A51.42 | Secondary syphilitic female pelvic disease |
| A51.43 | Secondary syphilitic oculopathy |
| A51.45 | Secondary syphilitic hepatitis |
| A51.49 | Other secondary syphilitic conditions |
| A51.5 | Early syphilis, latent |
| A51.9 | Early syphilis, unspecified |
| A52 | Late syphilis |
| A52.0 | Cardiovascular and cerebrovascular syphilis |
| A52.00 | Cardiovascular syphilis, unspecified |
| A52.03 | syphilitic endocarditis |
| A52.05 | Other cerebrovascular syphilis |
| A52.09 | Other cardiovascular syphilis |
| A52.1 | Symptomatic neurosyphilis |
| A52.11 | Tabes dorsalis |
| A52.12 | Other cerebrospinal syphilis |
| A52.13 | Late syphilitic meningitis |
| A52.16 | Charcot's arthropathy (tabetic) |
| A52.17 | General paresis |
| A52.3 | Neurosyphilis, unspecified |
| A52.31 | Condyloma latum |
| A52.7 | Other symptomatic late syphilis |
| A52.72 | Syphilis of lung and bronchus |
| A52.73 | Symptomatic late syphilis of other respiratory organs |
| A52.74 | Syphilis of liver and other viscera |
| A52.76 | Other genitourinary symptomatic late syphilis |
| A52.78 | Syphilis of other musculoskeletal tissue |
| A52.79 | Other symptomatic late syphilis |
| A52.8 | Late syphilis, latent |
| A52.9 | Late syphilis, unspecified |
| A53 | Other and unspecified syphilis |
| A53.0 | Latent syphilis, unspecified as early or late |
| A53.9 | Syphilis, unspecified |
